# An ontology-based approach to the analysis of the acid-base state of patients at operative measures

**DOI:** 10.7717/peerj-cs.777

**Published:** 2021-12-09

**Authors:** Man Tianxing, Mikhail Lushnov, Dmitry I. Ignatov, Yulia Alexandrovna Shichkina, Natalia Alexandrovna Zhukova, Alexander Ivanovich Vodyaho

**Affiliations:** 1 ITMO University, Saint Petersburg, Russia; 2 Almazov National Medical Research Centre, Saint. Petersburg, Russia; 3 National Research University Higher School of Economics, Moscow, Russia; 4 St. Petersburg State Electrotechnical University “LETI”, Saint Petersburg, Russia; 5 St. Petersburg Institute for Informatics and Automation of the Russian Academy of Sciences, Saint Petersburg, Russia

**Keywords:** Data mining, Acid-base state, Ontology, Semantic meta mining

## Abstract

Researchers working in various domains are focusing on extracting information from data sets by data mining techniques. However, data mining is a complicated task, including multiple complex processes, so that it is unfriendly to non-computer researchers. Due to the lack of experience, they cannot design suitable workflows that lead to satisfactory results. This article proposes an ontology-based approach to help users choose appropriate data mining techniques for analyzing domain data. By merging with domain ontology and extracting the corresponding sub-ontology based on the task requirements, an ontology oriented to a specific domain is generated that can be used for algorithm selection. Users can query for suitable algorithms according to the current data characteristics and task requirements step by step. We build a workflow to analyze the Acid-Base State of patients at operative measures based on the proposed approach and obtain appropriate conclusions.

## Introduction

There are currently millions of records accumulated in medical information systems (MIS) and databases. Data contains measurements of multiple parameters that reflect the state of the patients. Due to the fact that the human organism is a single system with multiple non-linear internal dependencies, there is a demand to analyze medical data from the physiological system positions (Osipov et al., 2017) and synergetics (Haken, 1977).

Data mining (DM) technology is used for revealing various non-linear dependencies and using them for solving applied problems. But despite the urgent need for system analysis of the organism state and the capabilities of DM, the existing DM models, methods, and algorithms are rarely used in practice. The problem that prevents using DM technique in the medical domain is that the raw data in real life is always complex and messy, researchers don’t know which method can be used to process it and often resort to trial and error. The rapid development of DM has led to the increase of the complexity of algorithm selection (Nalchigar, 2020). Traditionally, this kind of support is provided by experts/consultants. They are, however, often not available and also mired by the increasing number of algorithms.

**Figure 1:**
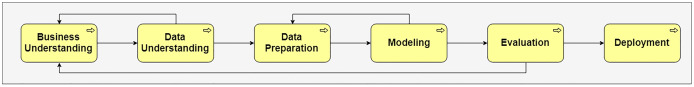
CRISP-DM model.

To construct and manage DM processes, cross-industry standard for data mining (CRISP-DM) was developed (Wirth & Hipp, 2000). It describes the typical DM processes, which are formalized within six stages with hundreds of activities (Fig. 1): business understanding, data understanding, data preparation, modeling, evaluation, and deployment. For the DM workflow construction, each stage requires the choice of the algorithms, thus, to get an effective solution we must spend many efforts and it takes a long time.

To build required processes for systematic medical data analyses, it is necessary to develop models, methods, and tools for DM workflow construction that takes into account particular features of data processing processes implemented in the medical domain and allow choosing DM methods and algorithms according to them.

Recently, many systems were proposed to support the DM processes: RapidMiner (Mierswa et al., 2006), OpenML (Vanschoren et al., 2014), Google’s prediction API (Bisong, 2019), Azure Machine Learning (Barnes, 2015), Weka (Holmes, Donkin & Witten, 1994), SAS (Allison, 2010), SPMF (Fournier-Viger et al., 2014), and (Volitich, 2008). These systems are developed based on the following novel techniques:

- Meta learning (Vanschoren, 2018), that is learning to learn, is defined as the application of machine learning (ML) techniques to meta-data about past machine learning experiments with the goal of modifying some aspects of the learning process in order to improve the performance of the resulting model.
- Semantic Meta Mining (SMM (Hilario et al., 2009)) is defined as DM process or workflow mining that is driven simultaneously by meta-data and by the collective expertise of data miners embodied in the DM ontology and knowledge base.
- AutoML (He, Zhao & Chu, 2021) is the process of automating the tasks of applying ML to real-world problems.

Meta learning and AutoML focus on data preparation and modeling stages of CRISP-DM. SMM supports the overall DM process by providing a suitable description framework to clarify the complicated relationship between tasks, data, and algorithms at different stages of the DM processes. The DM expertise is stored in a machine-interpretable format (ontology). However, most existing SMM systems only support solving general DM problems. Panov, Džeroski & Soldatova (2008) proposed a data mining ontology OntoDM, which includes formal definitions of basic DM entities. Then, Panov, Soldatova & Džeroski (2016) developed a separate ontology module, named OntoDT, for representing the knowledge about data types. Hilario et al. (2009) present the data mining optimization ontology (DMOP), which provides a unified conceptual framework for analyzing data mining tasks, algorithms, models, datasets, workflows, and performance metrics, as well as their relationships (Keet et al., 2015). There are several other data mining ontologies currently existing, such as the Knowledge Discovery (KD) Ontology (Žáková et al., 2010; Tianxing et al., 2019), the OntoDTA ontology (Benali & Rahal, 2017), the KDDONTO Ontology (Diamantini, Potena & Storti, 2009), the Data Mining Workflow (DMWF) Ontology (Kietz et al., 2009), which are based on similar ideas. These ontologies present the description of DM knowledge in general, for specific domain data they don’t provide targeted support. Thus, there is an urgent need to develop an approach to support the data analysis in specific domains, in particular, medical domain.

We propose an ontology-based approach to analyze data in medical domain. We solve the task of constructing a medical-oriented DM ontology to support algorithm selection by merging a DM ontology with a proper medical domain ontology. To reduce the complexity of querying the merged ontologies, we solve the task of extracting sub-ontologies that correspond to the tasks of data analyses solved by the users.

In this article, we consider the task of acid-base state (ABS) analysis of patients at operative measures. To characterize the organisms’ state, we consider the consistency of the parameters’ dynamics. To build the workflow for ABS analysis, we dynamically query on the ontology according to the data characteristics and task requirements at each stage of data analyses process and obtain suitable algorithms. To impute the raw data, the K-Nearest Neighbor algorithm (KNN) is chosen, and criteria functions (CF) (Narendra & Fukunaga, 1977) and functionals (Osipov et al., 2019) are selected to estimate the changes of organism parameters. The results of experimental research are given in the Results section. The “Materials and Methods” section presents the problem definitions, the details of the ontology-based system to support algorithm selection, and the workflow construction for the analysis of ABS of patients at operative measures. The “Results” section presents the conclusions of the analysis of ABS of patients. The “Discussion” section presents the evaluation of the proposed ontology-based approach. At last, the “Conclusion” section summarizes the results and benefits of this study.

## Materials and Methods

### Algorithm selection and workflow construction problem

The workflow construction problem is the problem of sub-processes selection and sequencing and algorithms selection for each sub-process. The DM workflows contain multiple processes that are decomposed into sub-processes. Workflow construction includes selecting the required sub-processes and sequencing them in a specific order. The corresponding algorithms are selected for each sub-process.

**Figure 2:**
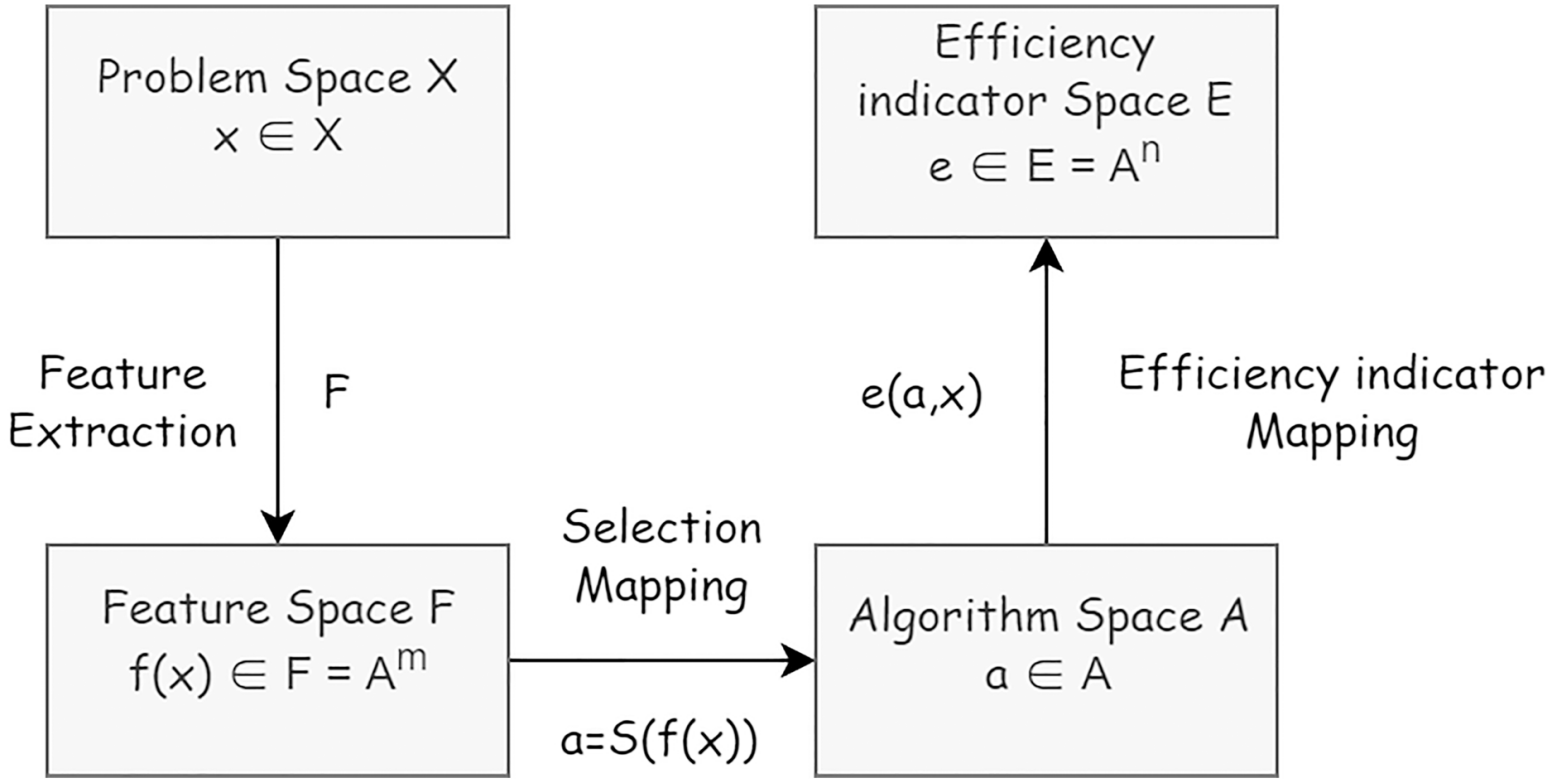
The formal model of algorithm problem.

The definition of the algorithm selection problem is based on the modified formal model (Rice, 1976). Within the algorithm selection problem the following elements are considered: a problem space *X* or collection of problem instances describable in terms of features defined in feature space *F*, an algorithm space *A* or set of algorithms considered to address problems in *X*, and an efficiency indicator space *E* representing metrics of workflow construction efficiency. An algorithm selection problem can then be formulated as follows: Given a problem $x \in X$ characterized by $f(x) \in F,$ find an algorithm $a \in A$ *via* the selection mapping *S*(*f*(*x*)) (Fig. 2).

To estimate the efficiency of data analyses workflows construction, the following efficiency indicators are considered:

- Completeness—whether the constructed workflow supports the overall DM process.
- Effectiveness–whether the usage of constructed workflows for data analyses provides the results that meet the requirements of the users.
- Performance–calculation time and calculation complexity of constructing workflows for data analyses.

### Definitions

The proposed approach is based on the notion of ontology. Generally, the ontology is composed of various components and represented as a five-tuple *O* = (*C*, *R*, *H*^*C*^, *H*^*R*^, *I*) Taye (2010), where:

- *C* represents a set of concepts (instances of “rdf:Class”). These concepts are arranged with a corresponding subsumption hierarchy *H*^*C*^.
- *R* represents a set of relations that relate concepts to one another (instances of “rdf:Property”). *R*_*i*_ ∈ *R* and *R*_*i*_ $\subseteq C \times C.
- *H*^*C*^ represents a concept hierarchy in the form of a relation (a binary relation corresponding to “rdfs:subClassOf”). ${H^C} \subseteq C\times C$, where *H*^*C*^(*C*_1_, *C*_2_) denotes that *C*_1_ is a sub-concept of *C*_2_.
- *H*^*R*^ represents a relation hierarchy in the form of a relation ${H^R} \subseteq R \times R$, where *H*^*R*^(*R*_1_, *R*_2_) denotes that *R*_1_ is a sub-relation of *R*_2_ (“rdfs:subPropertyOf”).
- *I* is the instantiation of the concepts in a particular domain (“rdf:type”).

In RDF(S) (Resource Description Framework Schema) statements, the content has a unified form: subject, predicate, object. The entities for subject and object are classes, datatypes and individuals. The entities for predicate are object properties and data properties. Thus, we can formalize the ontology *O* as a two-tuple: *O* = {*C*, *R*}, where: *C* is the set of subjects and objects; *R* is the set of predicates. This simple assertion model is an information network. Therefore, we can intuitively understand the collection of information resources and RDF(S) sentences and describe them as graphs.

### An ontology-based system to support algorithm selection

**Figure 3:**
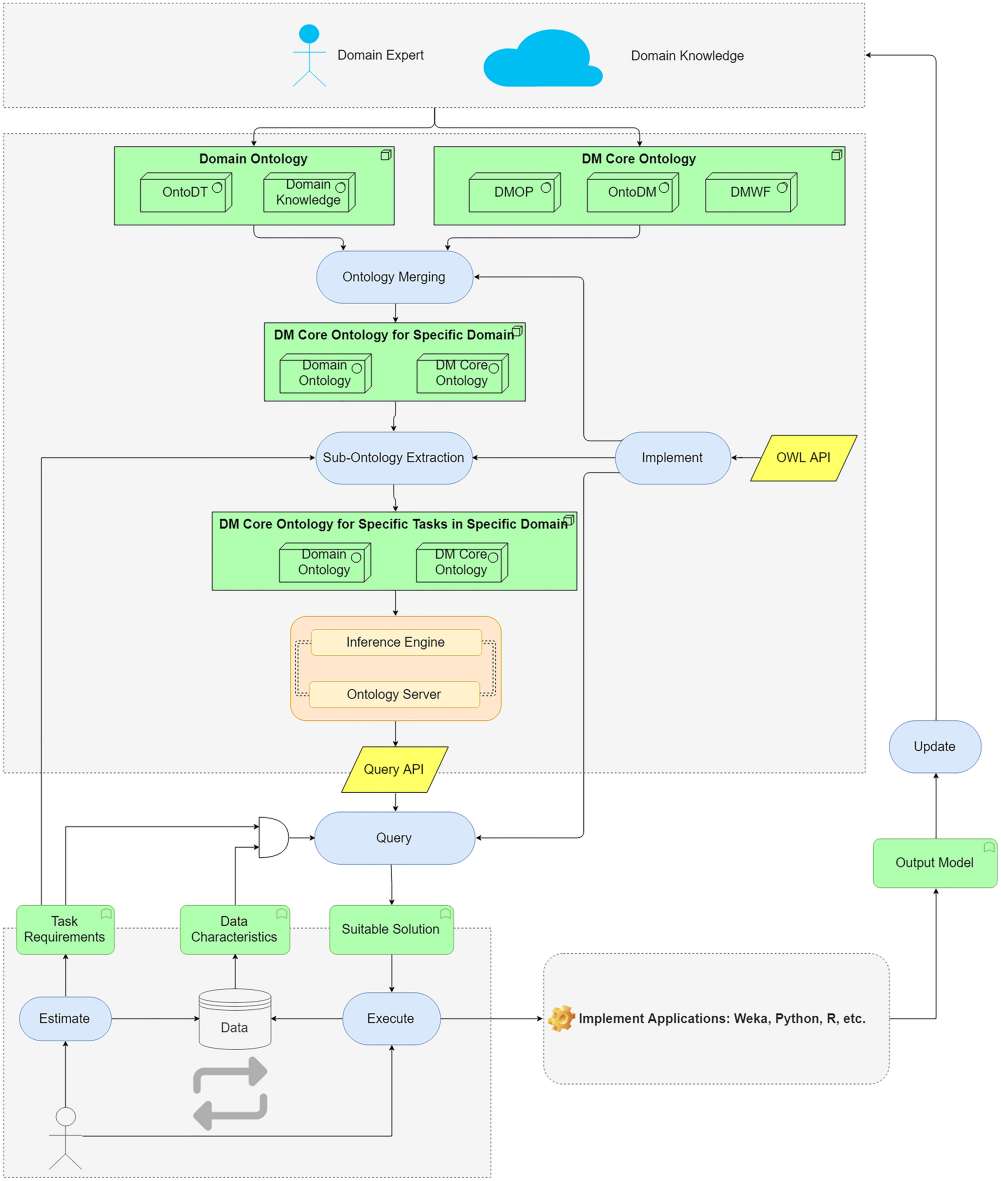
The main components of the proposed ontology-based approach.

We propose an ontology-based approach to help users to query for suitable DM methods and algorithms. The main components of the approach are shown in Fig. 3.

The domain data characteristics are described in domain ontology and DM Core ontology describes DM algorithms and their characteristics. The ontologies are merged to map domain knowledge and DM knowledge. The merged ontologies are large-scaled ontologies as they contain redundant DM knowledge for a specific domain. By an extraction method, the sub-ontology corresponding to a specific task requirement is extracted. The generated ontology is used as a knowledge base to support algorithms selection. The inference engine and ontology server implement the query API to the knowledge base. By building queries, users can search for the suitable DM algorithms according to the task requirements and data characteristics. The generated workflows are converted to the readable files for the DM tools. Then, users can perform the workflows in the operating environments (Python, R, etc.) to analyze domain data.

#### Design of DM core ontology

**Figure 4:**
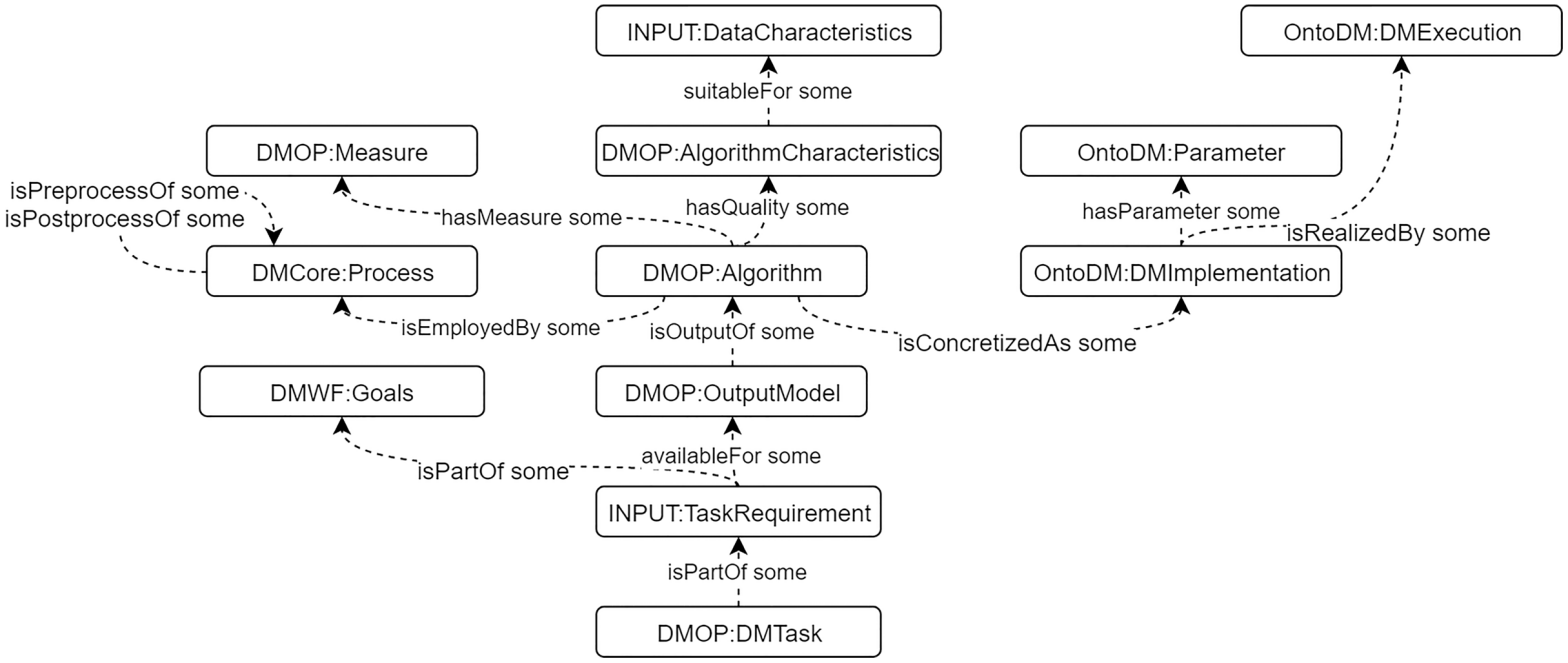
The main classes and properties of DM Core ontology.

We built a DM Core ontology to describe general DM knowledge, including algorithms, processes, characteristics, goals, etc. The construction is based on CRISP-DM model (Wirth & Hipp, 2000). Some existing DM ontologies are integrated, such as OntoDM (Panov, Džeroski & Soldatova, 2008), DMOP (Keet et al., 2015), and DMWF (Kietz et al., 2009). We also define and use the “INPUT” sub-ontology to describe the data characteristics and task requirements to support algorithms selection. The detailed description of the DM Core ontology is presented in (Tianxing et al., 2020). The main classes and properties are presented in Fig. 4.

#### Ontology merging method

The general characteristics of the data sets and task requirements that are defined in DM Core ontology are the basis for algorithm selection. They generalize the characteristics used in different domains that are defined according to different standards. Domain experts define these characteristics based on the attributes of the initial data sets using their knowledge. Merging method allows import them into DM ontology and thus generate the merged ontology as a domain-oriented DM ontology (Ganter & Stumme, 2003).

**Figure 5:**
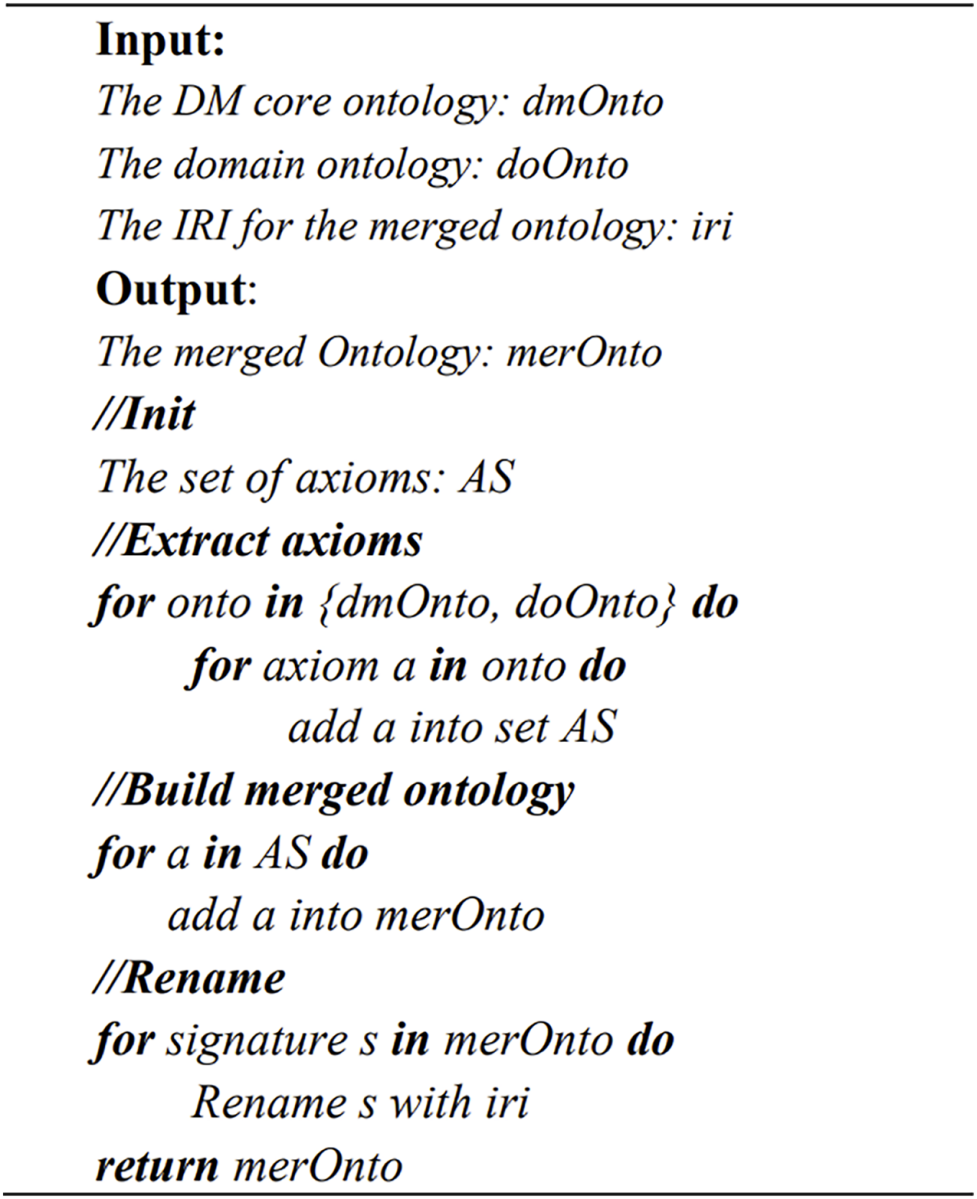
The pseudo code of ontology merging method.

The merging module is implemented based on the OWLAPI interface (Horridge & Bechhofer, 2011). The main idea is to extract all the axioms in both ontologies and then save and rename the IRI in a newly merged ontology (Fig. 5).

#### Sub-ontology extraction method

The size of the merged ontologies is often large, so queries have to take a long time. In order to improve the efficiency of the queries execution, we propose an ontology extraction method based on the restriction path. The restriction path is a path from the bottom of the ontology to its super classes. The method allows extracting the task-related sub-ontology from DM ontology in a bottom-up way. By reducing the size of the ontologies, the complexity of ontology querying is reduced.

**Figure 6:**
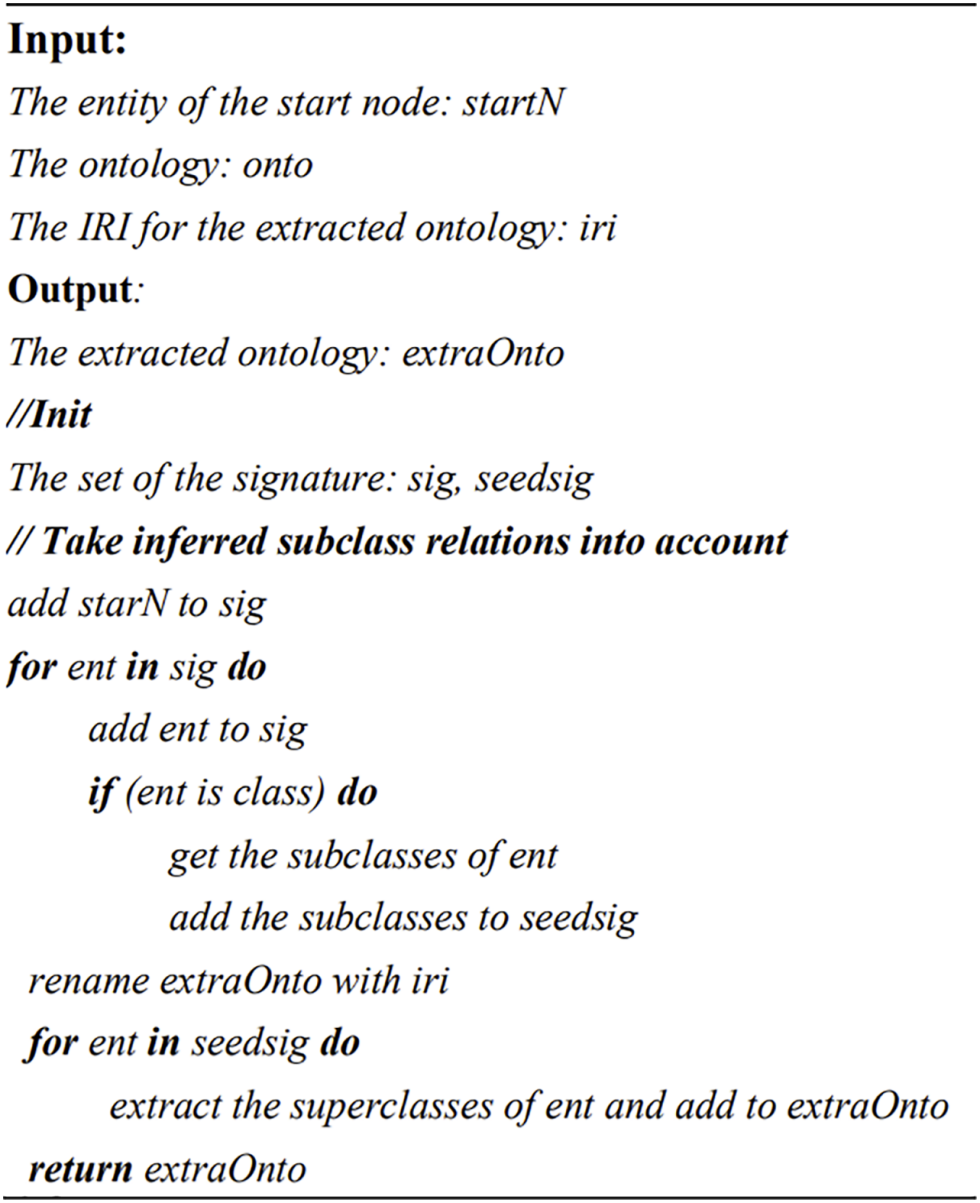
The pseudo code of sub-ontology extraction method. IRI (Internationalized Resource Identifier) is an ontology IRI.

The main idea of the proposed extraction method is to extract the content along a path in a one-way directed graph. The input of this method is the DM ontology and the task requirement (which is an entity of the ontology), and the output is a sub-ontology for solving a specific task. The extraction module is also implemented based on the operators in OWLAPI, which is shown in Fig. 6.

#### Inference engine and ontology server

The Description Logic (DL) query is a class expression that is defined using constructs such as “and” and “some” and so on. It allows users quickly get definitions of classes and other information, in particular, subclasses that they subsume (Horridge & Patel-Schneider, 2009).

Due to the ease of understanding of the DL queries, we choose it as the way to query the ontology. A DL query tab was developed as a part of Protégé ontology editor. The query language supported by the tab is based on the Manchester OWL syntax that enables collecting all the information about a particular class, property, or individual into a single construct (Horridge & Patel-Schneider, 2008).

The DL query is performed on an ontology classified by a reasoner (inference engine). In Protégé Hermit reasoner (Glimm et al., 2014) is employed.

Users can query for suitable algorithms according to the task requirements and data characteristics. An example of DL query is as following:

*Q*: *Which algorithms are suitable for small size datasets*?

*DL query*: *Algorithm and suitableFor some SmallSizeDataset*

Users perform the DL queries on the extracted sub-ontology to construct the workflow by the following steps:

- Query for the general DM stages (the six CRISP-DM stages).
- Query for the available processes.
- Query for the suitable processes for each stage.
- Query for the suitable operator/algorithm for each process.

The query-execute-estimate process is a cyclic process. By applying the selected algorithm, the data characteristics are updated. Based on the data’s current state, we estimate whether the data is ready for the next processing stage. If not, we need to continue selecting the corresponding data preprocessing algorithm.

### Analysis of acid-base state of patients at operative measures

For constructing the suitable workflow, we follow the general DM process (presented in Fig. 3) defined in the ontology. Five stages (Task understanding, Data understanding, Data preparation, Data processing, and Evaluation) are the main stages of the data analyses process based on the CRISP-DM model that starts with getting an understanding of the task. An understanding of the data is obtained, and then, the data is preprocessed for the further data processing stage where the actual DM algorithms are defined. The output model generated by data processing algorithms is then analyzed by researchers to obtain the conclusions.

The DM workflows construction using ontologies is based on the following components:

- The defined sequence of the DM processes in CRISP-DM are presented with property “hasPostprocess” in the DM ontology. For example, the sequence of the general DM stages is defined and shown in Fig. 1.
- By specifying the input and output of the processes as the restrictions, the sequence of the DM processes is determined. For example, the process “MissingValueProcessing” has input “DataWithMissingValue” and output “DataWithoutMissingValue”, then, it should be before the process “Modelling”, which has input “DataWithoutMissingValue” and output “OutputModel.”

#### Ontology merging and extraction for medical domain

**Figure 7:**
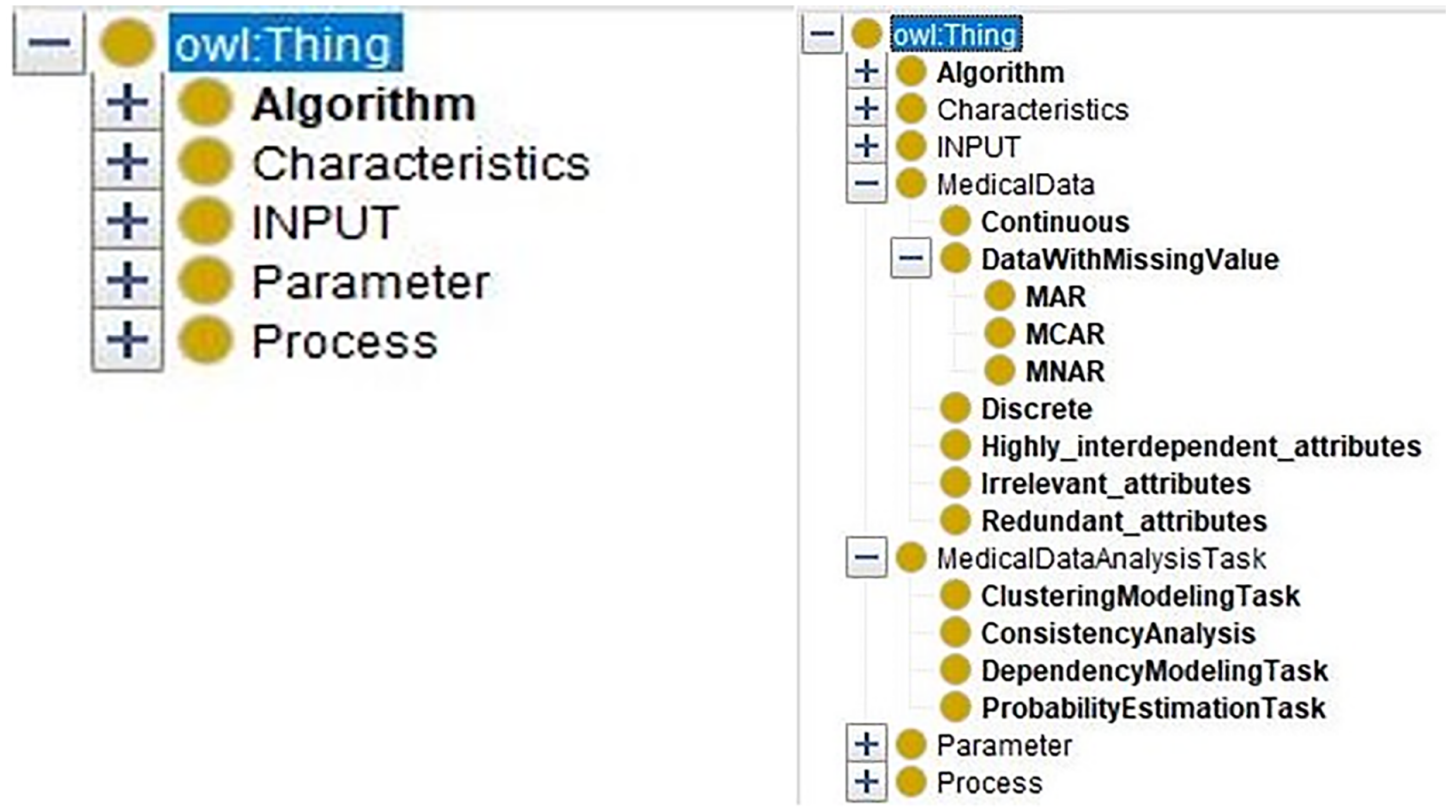
The comparison of the DM Ontology and the merged ontology.

At first, the data characteristics and the medical data analysis tasks are defined in the medical domain ontology. The merged ontology (Fig. 7, right part) has the entities from domain ontology “MedicalDataAnalysisTask, MedicalData” and the entities from DM ontology (Fig. 7, left part) “Algorithm, Characteristics, INPUT, Parameter, and Process.”

In sub-ontology extraction module, users input the task requirements and extract the sub-ontology which is related to the task requirements.

**Figure 8:**
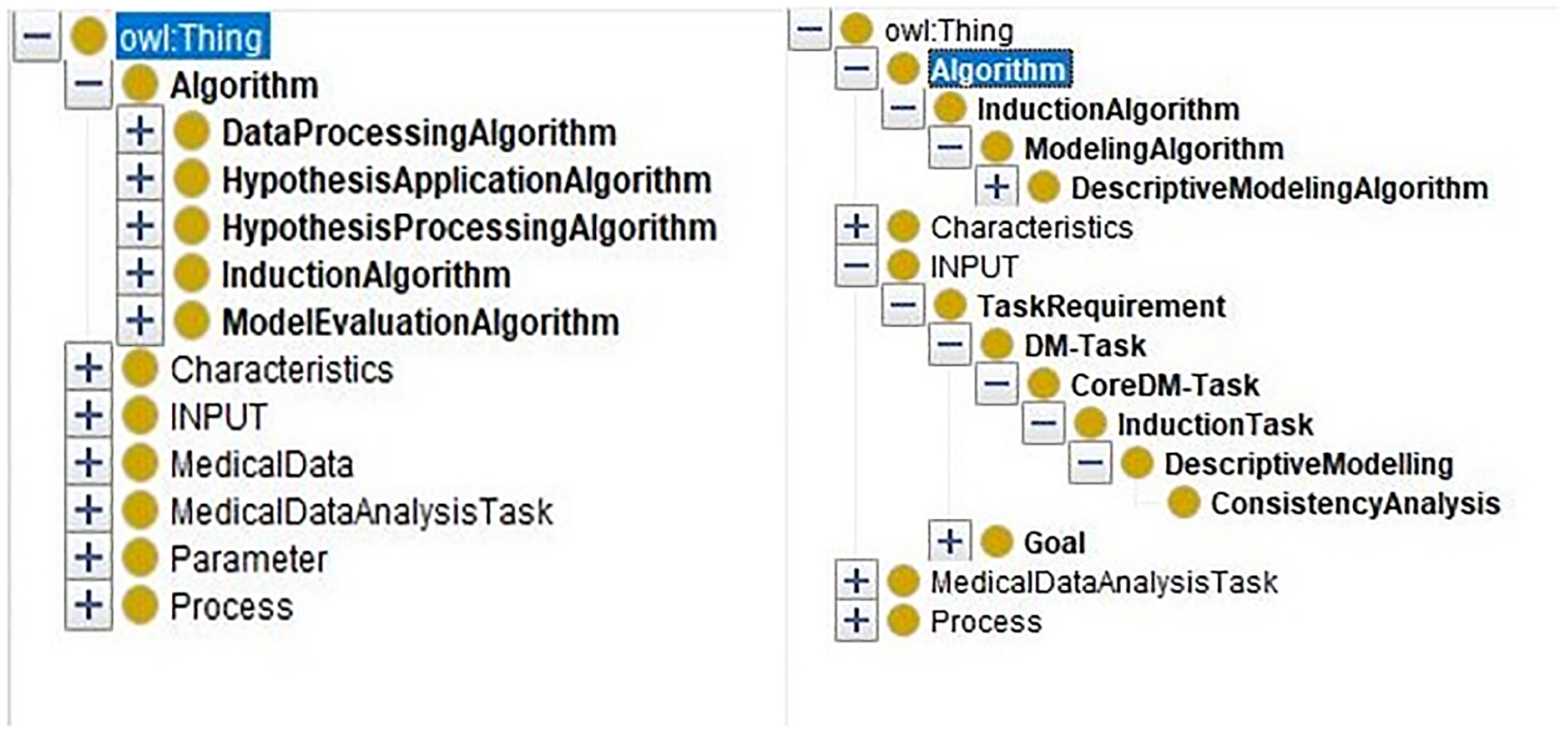
The comparison of the merged ontology and the extracted ontology.

By inputting the task requirement “ConsistencyAnalysis”, we can extract the sub-ontology for solving the consistency analysis task from the merged ontology. Only related entities are kept. In Fig. 8, the comparison of the merged ontology (left part) and the extracted sub-ontology (right part) is shown. For example, the merged ontology has kinds of induction algorithms, such as “classificationModelingAlgorithms”, “ClusteringModelingAlgorithm”, and so on. However, these algorithms are not employed by “ConsistencyAnalysis” task besides the “DescriptiveModelingAlgorithm”. Thus, only the descriptive algorithms are extracted and presented in the extracted sub-ontology (right part).

#### Task understanding

**Figure 9:**
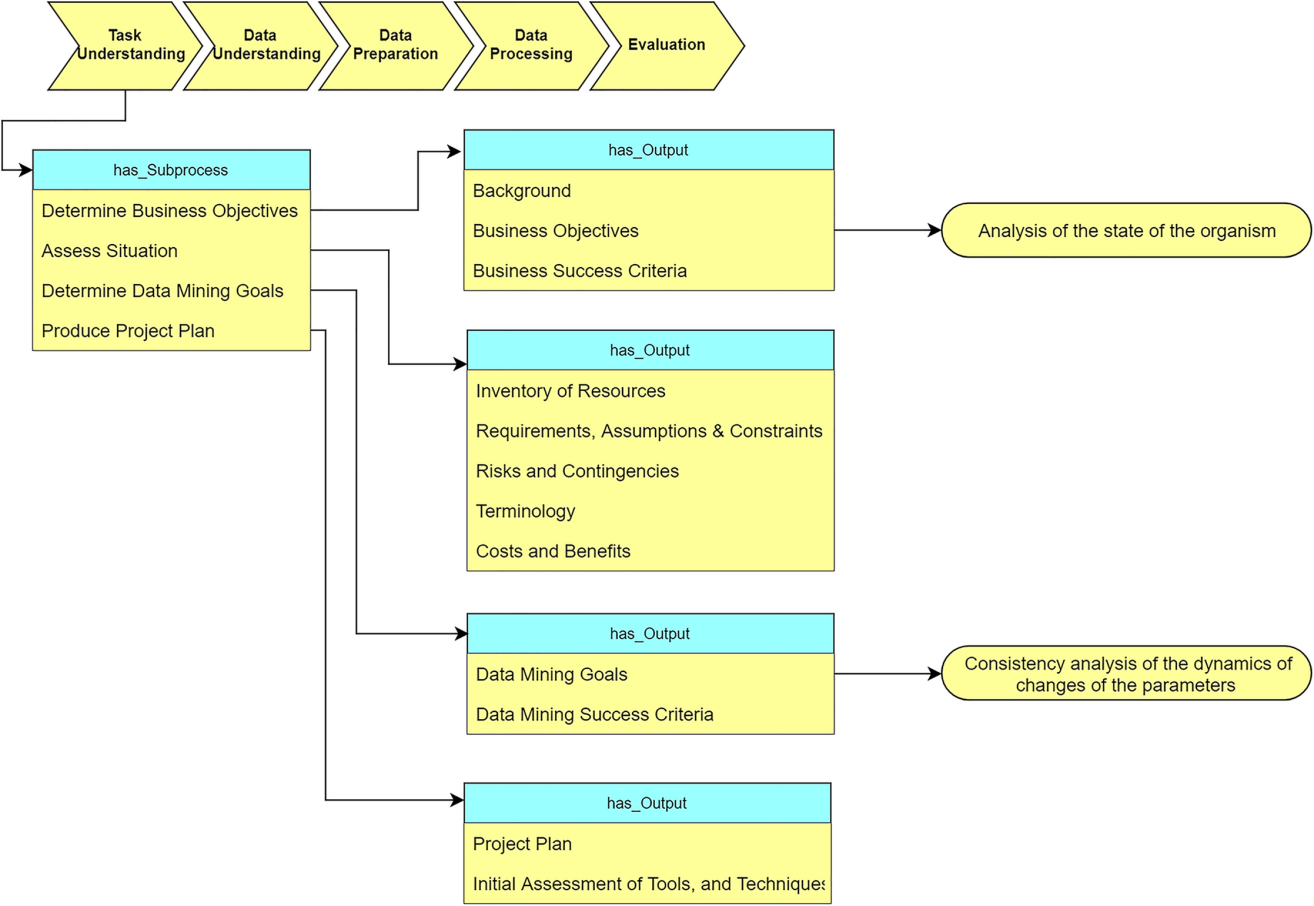
Query at the “Task understanding” stage (The yellow rectangle represents classes in the ontology, the blue rectangle represents object properties, the green rectangle represents data properties, and the red rectangle represents data types. In the subsequent figures the same colors are used).

As Fig. 9 shows, the “Task understanding” stage defines the sub-processes and corresponding output for analysis of the tasks. We determine our task objective as “analysis of the state of the organism.” Based on this requirement, we convert the task objective to DM objective “analysis of the consistency of the dynamics of changes of the parameters”.

#### Data understanding

**Figure 10:**
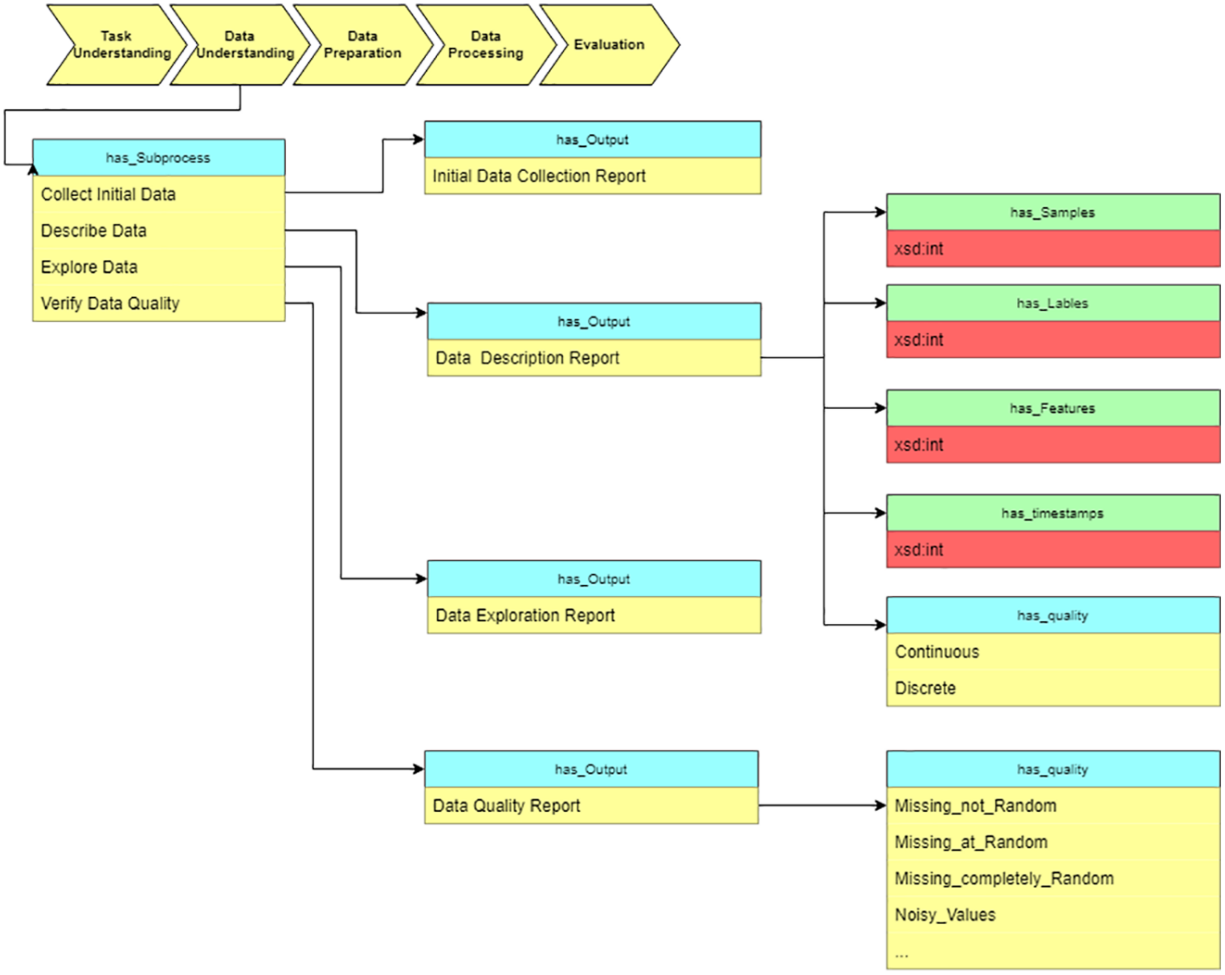
Query at the “Data understanding” stage.

After determining the objective, we move to the “data understanding” stage and estimate the data characteristics. As Fig. 10 shows, the description report contains the data’s metadata. Data properties (has_Samples, has_Labels, etc.) defined in the ontology assume description of the value and range of the basic dataset information.

As a sub-process, we verify the data quality to estimate whether the data meets the requirements of data processing algorithms. The output of this stage is used as input for the next stage, “Data preparation.” The “Data description report” and “Data quality report” provide the basis for choosing data preprocessing algorithms.

#### Data preparation

**Figure 11:**
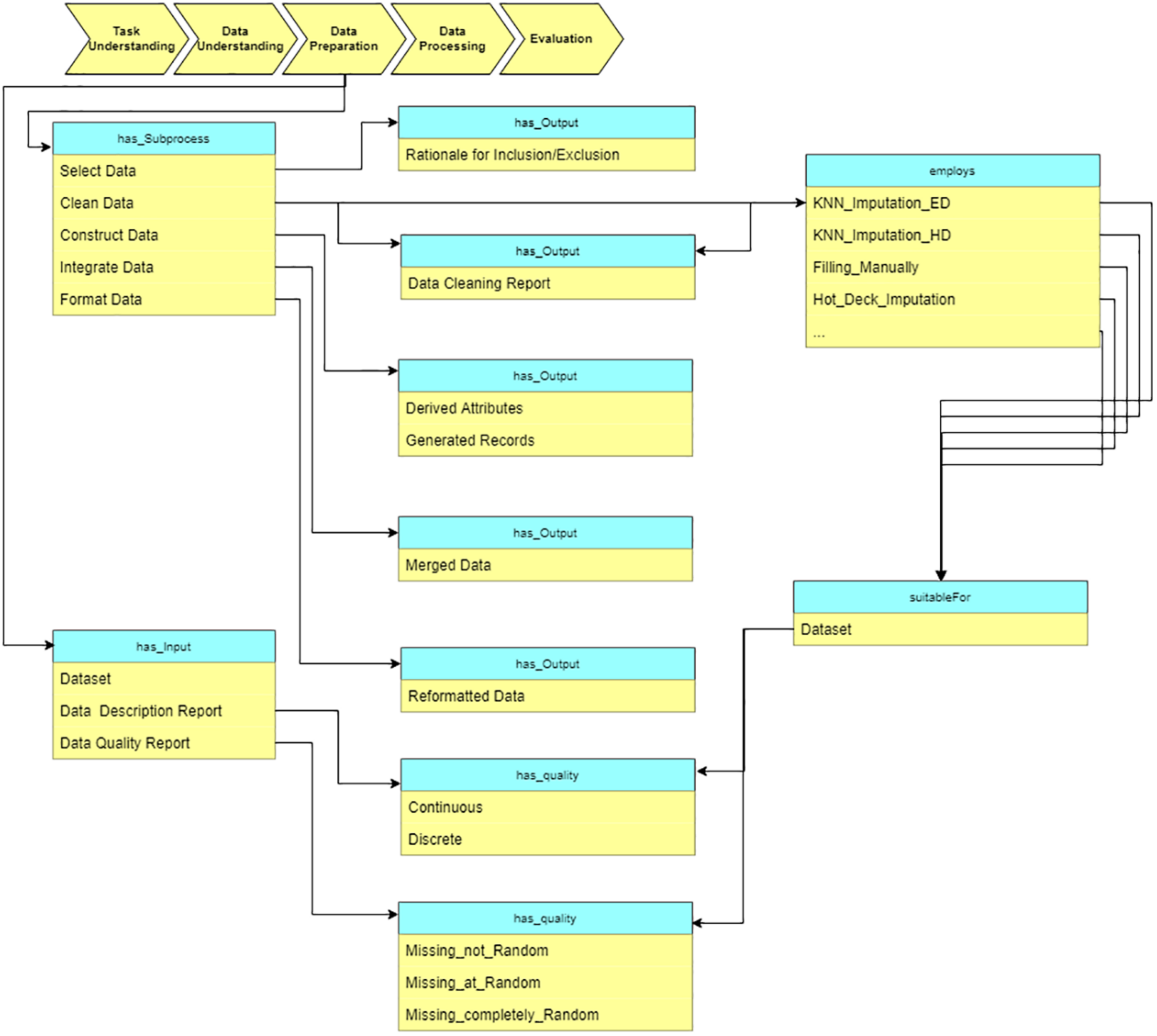
Query at the “Data preparation” stage.

At the “Data preparation” stage, we preprocess the raw data based on the reports from the “Data understanding” stage. The sub-processes are: select data, clean data, construct data, integrate data, and format data (see Fig. 11). As an example, some missing value imputation algorithms are presented. Each of them is suitable for a specific situation (which is defined in terms of specific data characteristics). These characteristics are obtained at “Data understanding” stage.

#### Data processing

**Figure 12:**
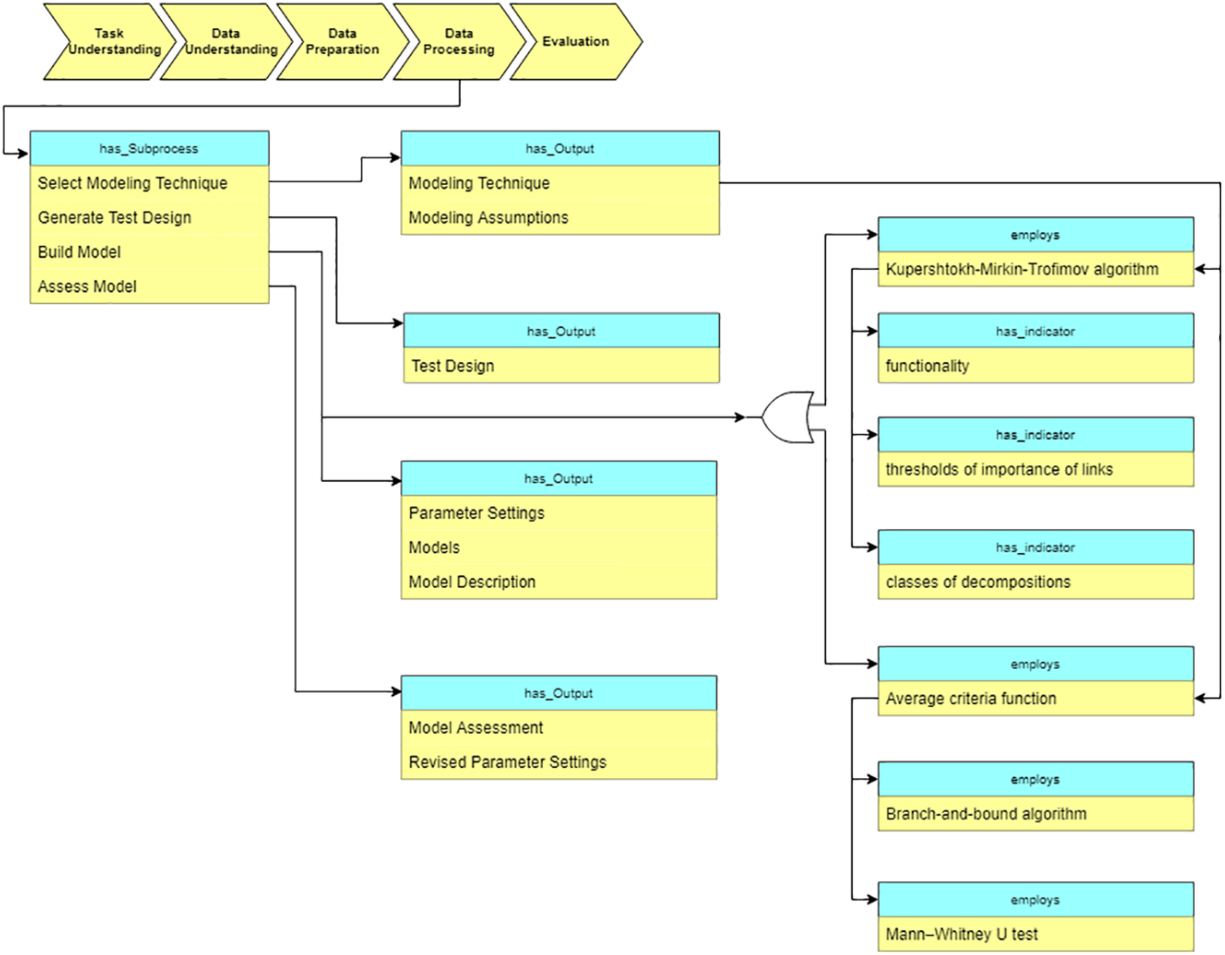
Query at the “Data preparation” stage.

At the “Data processing” stage, the data processing algorithms are selected based on the data characteristics and the task requirements. Besides the traditional DM algorithms, we also describe various domain specific methods in the ontology. In particular, for the analysis of cardiological patients’ data, the following algorithms are included into the ontology (see Fig. 12): Kupershtokh–Mirkin–Trofimov algorithm (Kupershtokh, Mirkin & Trofimov, 1976) and average Criteria Functions (CF) (Narendra & Fukunaga, 1977).

As Fig. 12 shows, CF is calculated based on the results of separate parameters measurements, which characterize the state of the organism as a system. We construct correlation matrix for the parameters of the organism state and evaluate the results using criteria (Chen, 2003).

It can be done using the branches and bounds procedure with the choice of the optimal subset of characteristics and evaluation of the criteria function for each patient. The method is based on the assessment of a monotonous function, which is CF from any biological set *A*. The algorithm is based on the calculation of the maximum CF on the basis of a certain quadratic form and on the search of the biggest set of *n* variables maximizing CF for all subsets containing *m* features. CF is calculated through a quadratic form:

$C({A_m}) = (X_m^T)S_m^{ - 1}({X_m})$

where:

- *A*_*m*_ is the set *m* of variables,
- *X*_*m*_ is a vector of variables (a set of bioparameters, which is the functional system of the specific patient)
- *S*_*m*_ is the symmetric positive correlation matrix of the size *m × m*; $X_m^T$ means transposing operation of a vector *X_m_*
-
$S_m^{ - 1}$ is the inverse matrix of *S_m_*.

Another supported algorithm is the Kupershtokh–Mirkin–Trofimov algorithm (Kupershtokh, Mirkin & Trofimov, 1976), which is based on the search of decomposition of sets of objects into disjoint classes. As Fig. 12 shows, three indicators are considered in this case: functionality, a threshold of the importance of links, and the number of classes of decomposition. We calculate and analyze such indicators for overall data, and then identify critical periods in the system (Lushnov et al., 2016).

The method is based on the calculation of an integral indicator, also known as an electrolyte balance functional. The idea of calculating this indicator is shown below. The set of objects (biochemical parameters, ions of blood, etc.) *R* = (*R*_1_, *R*_2_, …, *R*_*m*_) is divided into disjoint classes—sets of functional subsystem of physical parameters, delivering local maximum of the functional. Functional is described by the sum of correlation links between parameters subtracted by a certain threshold, characterizing significance of the correlation links, according to:

$F(a,R) = \sum\limits_s \sum\limits_{i,j \in {R_s}} ({a_{ij}} - a)$

where

- *a* is a link threshold (*a*_*ij*_ > *a* means the link is significant).
- *a*_*ij*_ < *a* means the link is not significant, *a_ij_* is the link indicator between *i* and *j* parameters (*a*_*ij*_ = *a*_*ji*_ and *a*_*ii*_ are not considered).
- *i*, *j* ∈ *R*_*s*_ means parameters *i* and *j* are included into *R*_*s*_ class.

#### Evaluation

**Figure 13:**
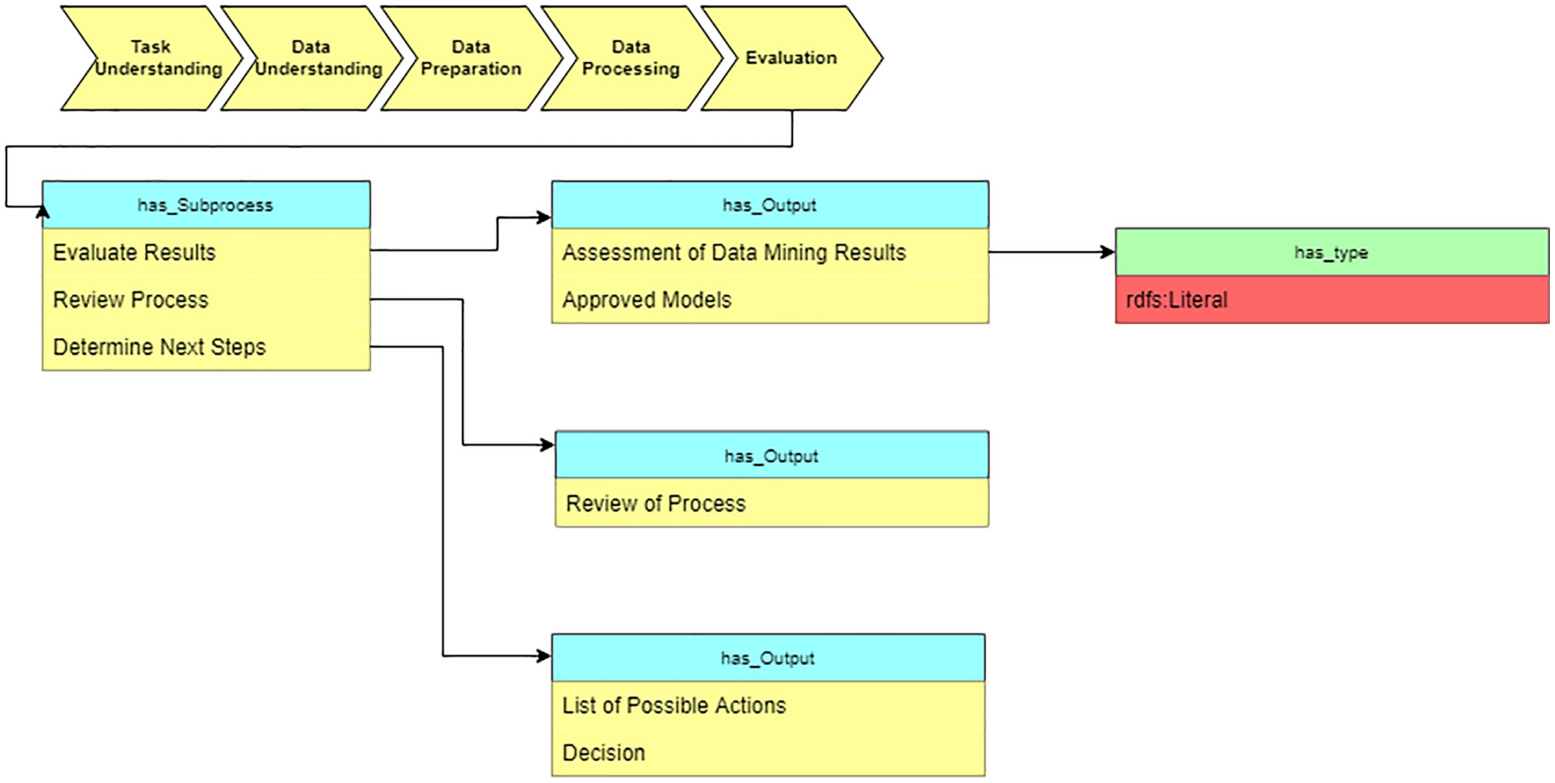
Query at the “Evaluation” stage.

In the “Evaluation” stage, we summarize the results of applying suggested algorithms on medical datasets (see Fig. 13).

## Results

For experimental research of medical data, the ontology-based system is developed based on OWLAPI interface (Horridge & Bechhofer, 2011) that is available at https://github.com/mantx626/ABS_Analysis.git. The main dependencies in the project are JUnit 4.12, owlapi 5.0.0, and hermit 1.3.8.500. The DL query program and reasoner are also embedded in this project. Users can query for suitable DM solutions using this project or Protégé software.

To estimate the proposed approach we collected the parameters of the ABS of three groups of patients at operative measures (Kurapeev & Kabanov, 2020). Data was collected by the authors and approved by the ethics committee of the Almazov National Medical Research Centre for our research. We characterize the state of the organisms based on the consistency of the dynamics of parameters’ changes. The raw data is the dynamics records of the ABS in cavernous sinus (CS) for 391 patients in total with cardiac surgical pathology during the postoperative period in the operating room and in the cardio-resuscitation unit at six points. There are 21 parameters of the ABS, which are listed in Table 1.

**Table 1:** The parameters of the ABS.

Parameter name	Description of the parameter	Parameter name	Description of the parameter
pH	Acidity	Na+	Sodium ion concentration
pO2	Oxygen partial pressure	Ca++	Calcium ion concentration
pCO2	Carbon dioxide partial pressure	Cl−	Chlorine ion concentration
ABE	Excess base	Glu	Glucose concentration
SBE	Lack of reason	Lac	Lactate content
cHCO3	Plasma bicarbonate	p50	Hemoglobin affinity for oxygen
cHCO3-st	Bicarbonate (alkali)	mOsm	Blood osmolarity
sO2	Oxygen boost	pH(T)	Acidity corrected for temperature
ctHb	Reference hemoglobin level	pO2(T)	Partial oxygen pressure adjusted for temperature
Htc	Hematocrit	pCO2(T)	Carbon dioxide partial pressure adjusted for temperature
K+	Potassium ion concentration		

**Figure 14:**
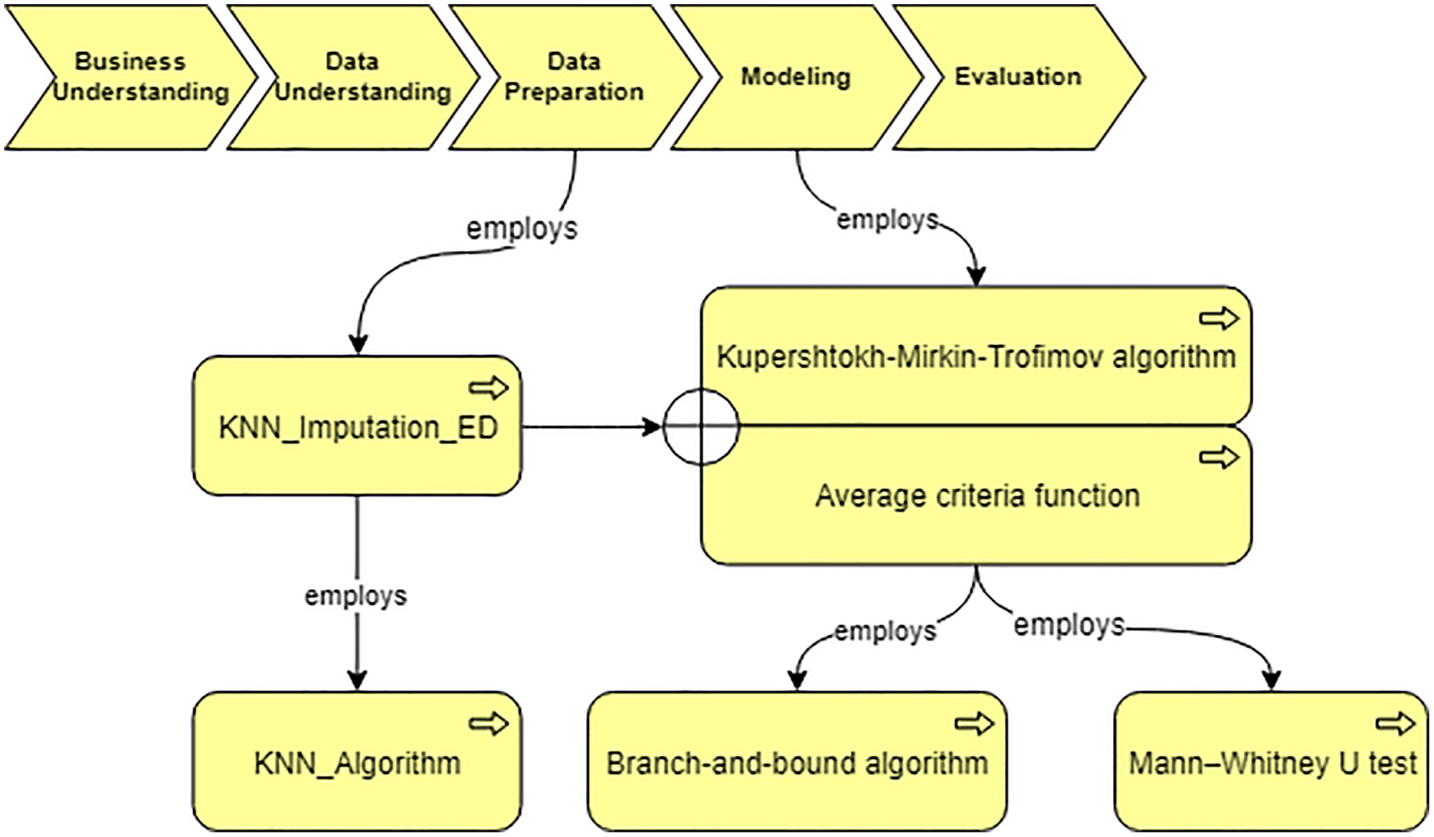
The generated workflow and the selected algorithms.

**Figure 15:**
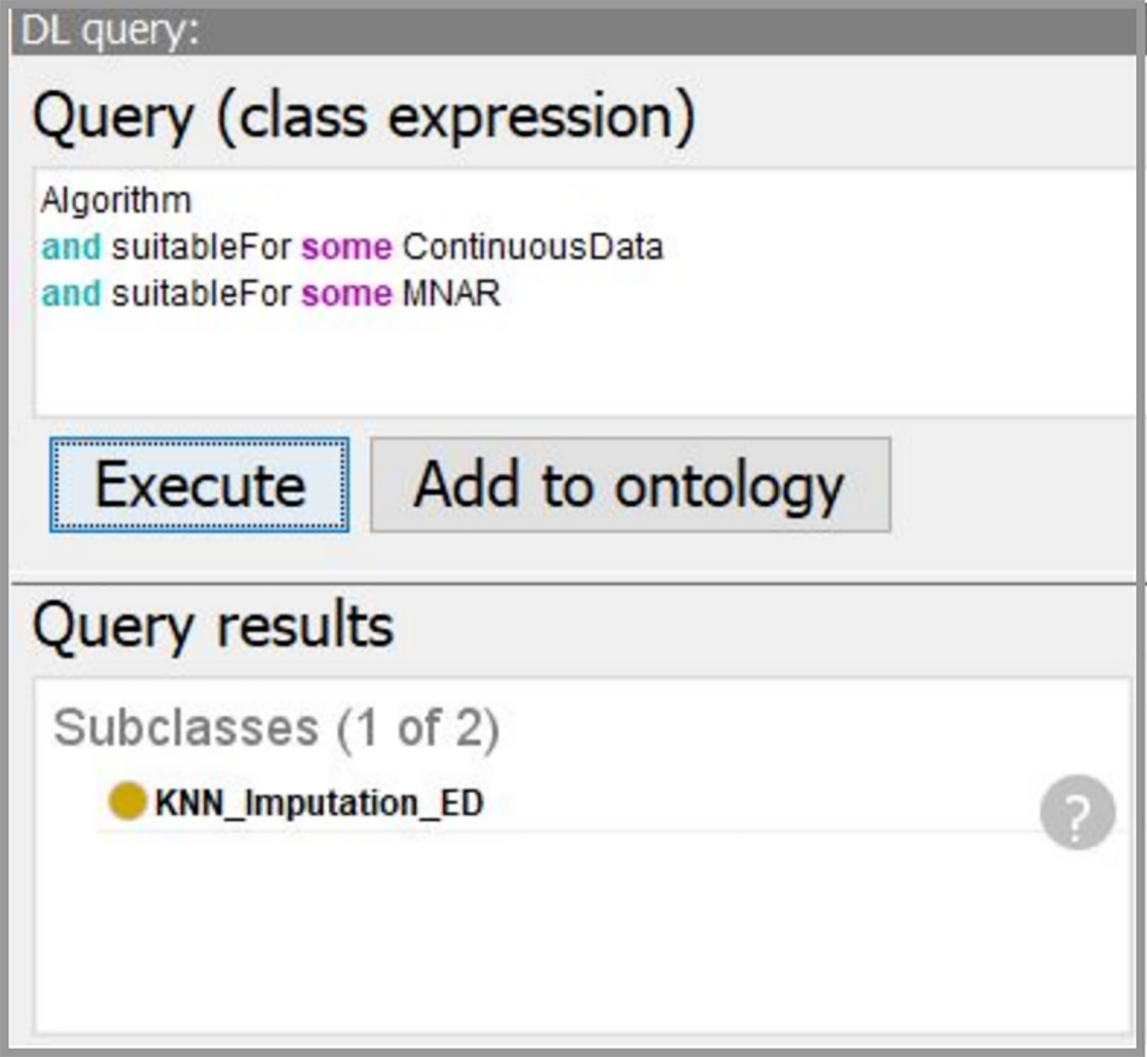
The screenshot of querying for suitable imputation algorithms.

**Figure 16:**
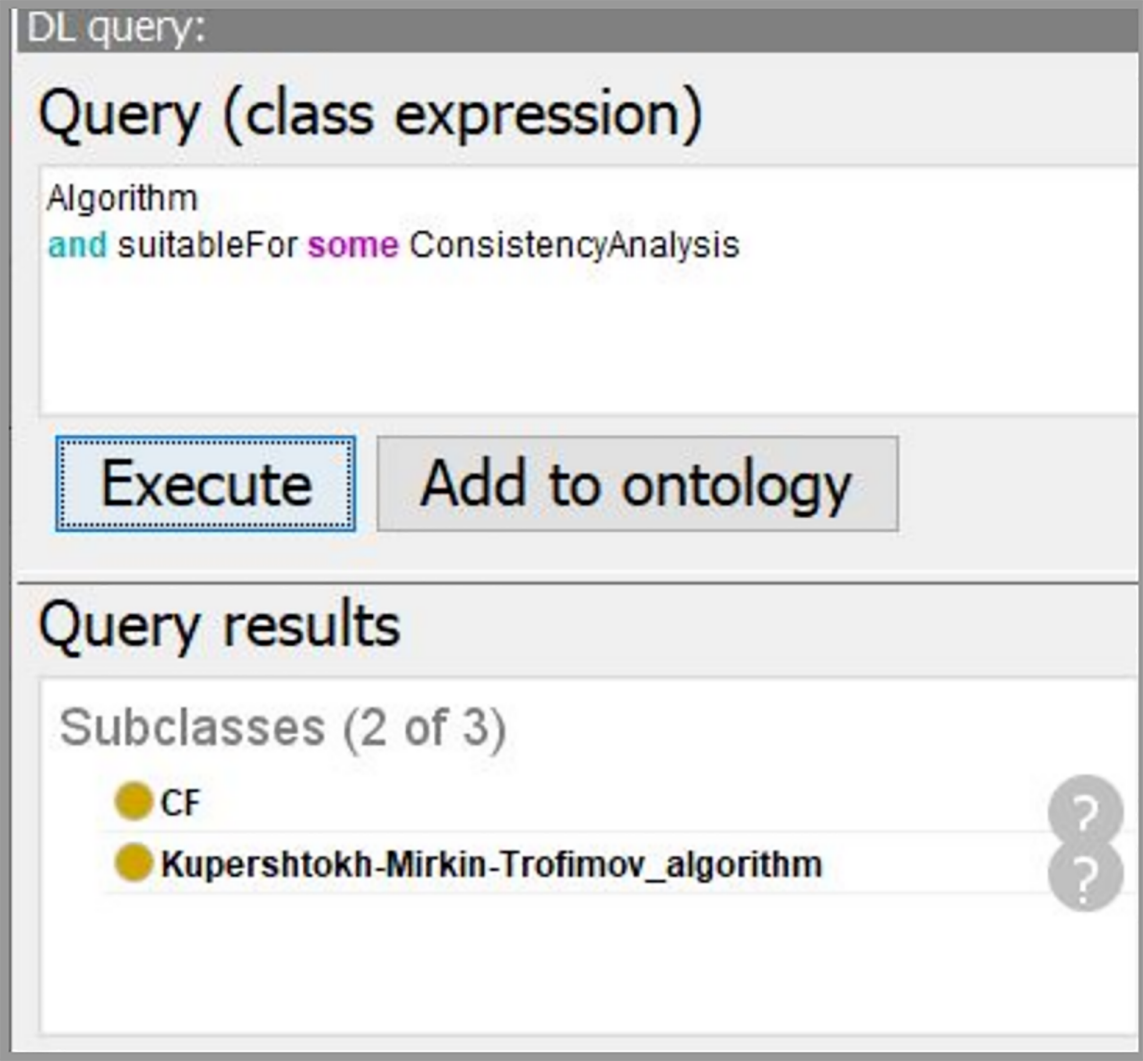
The screenshot of querying for suitable DM algorithms.

Using the developed system, we obtain the workflow that is presented in Fig. 14. In our case, the data characteristics “Continuous” and “Missing_not_Random” are obtained on “Data Understanding” stage. Thus, we query for a suitable algorithm to fill the missing values and the ontology suggests us to use “KNN_Imputation_ED”. The screenshot of querying for suitable imputation algorithm is presented in Fig. 15. The selected algorithm “KNN_Imputation_ED” is presented as an algorithm that employs “KNN_Algorithm (K_Nearest_Neighbor Algorithm)” and has measure “Euclidean_Distance.” For data processing, the ontology proposes to apply two algorithms, CF and Kupershtokh–Mirkin–Trofimov algorithm, according to the task requirement “consistency analysis”. The screenshot of querying for suitable DM algorithms is presented in Fig. 16.

**Figure 17:**
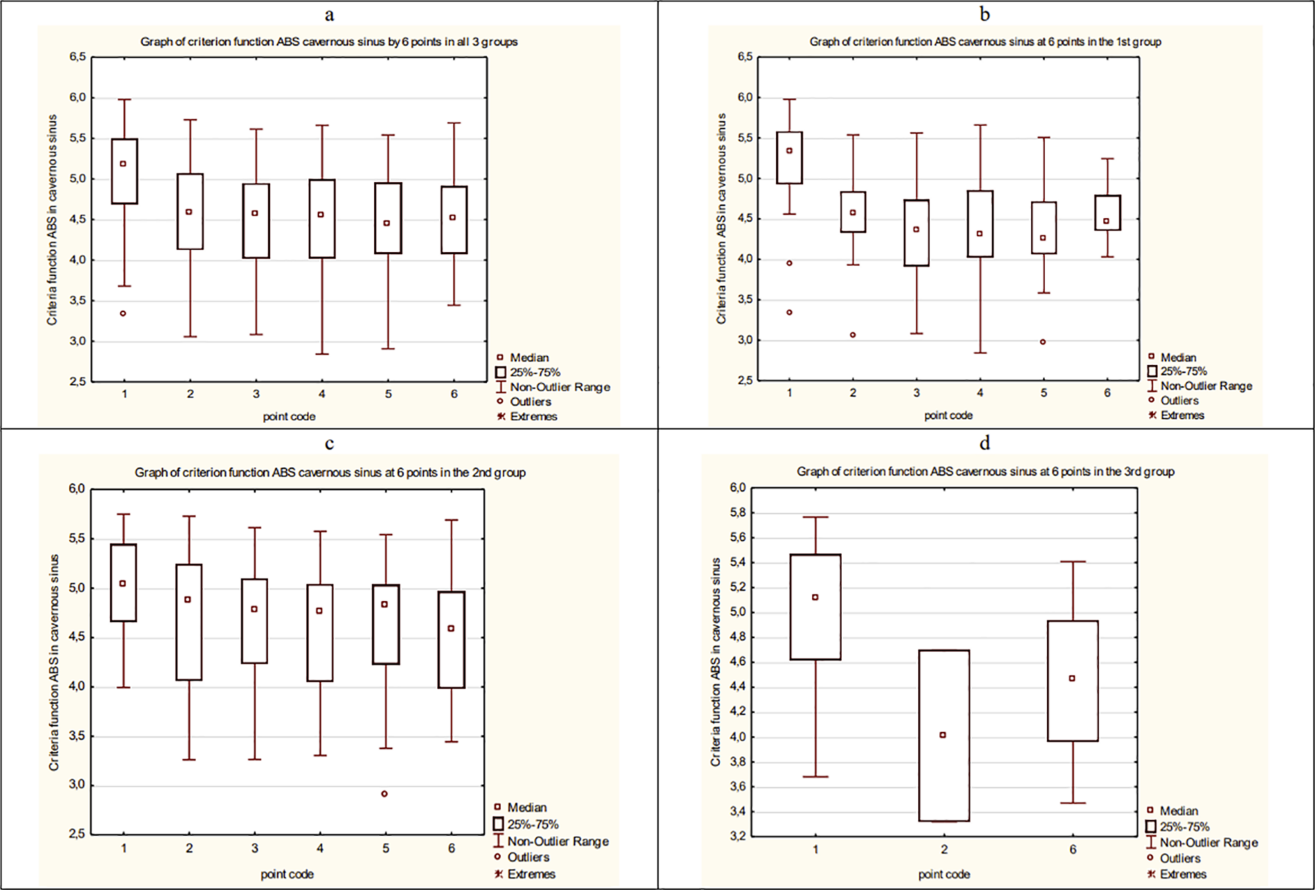
The average dynamics of CF of ABS in overall data and each group of patients. (A) The average dynamics of CF of ABS in a cavernous sinus in three groups. (B) The average dynamics of CF of ABS in a cavernous sinus in the 1st group. (C) The average dynamics of CF of ABS in a cavernous sinus in the 2nd group. (D) The average dynamics of CF of ABS in a cavernous sinus in the 3rd group.

As a result, the average dynamics of CF of ABS in overall data and each group of patients is estimated. The results are presented in Fig. 17.

Then, we compare and analyze the result using Mann–Whitney standard non-parametric criteria. The following conclusions about the dynamic of ABS of patients in CS can be made:

- CF averages for the group values of ABS between the 1st and 2nd groups differ significantly.
- The CF average values of ABS in CS between 1 point and 2, 3, 4, 5, 6 points in all three groups are significantly different in pairs.
- The CF average values of ABS in CS between 1 point and 2, 3, 4, 5, 6 points in the 1st group are significantly different in pairs.
- The CF average values of ABS in CS between 1 point and 2, 3, 4, 5, 6 points in the 2nd group are significantly different in pairs.
- The CF average values of ABS in CS between 1 point and 6 point in the 3rd group are significantly different.
- In the 3rd group, CF values of ABS in CS between the 1st and 2nd points are significantly different.

**Figure 18:**
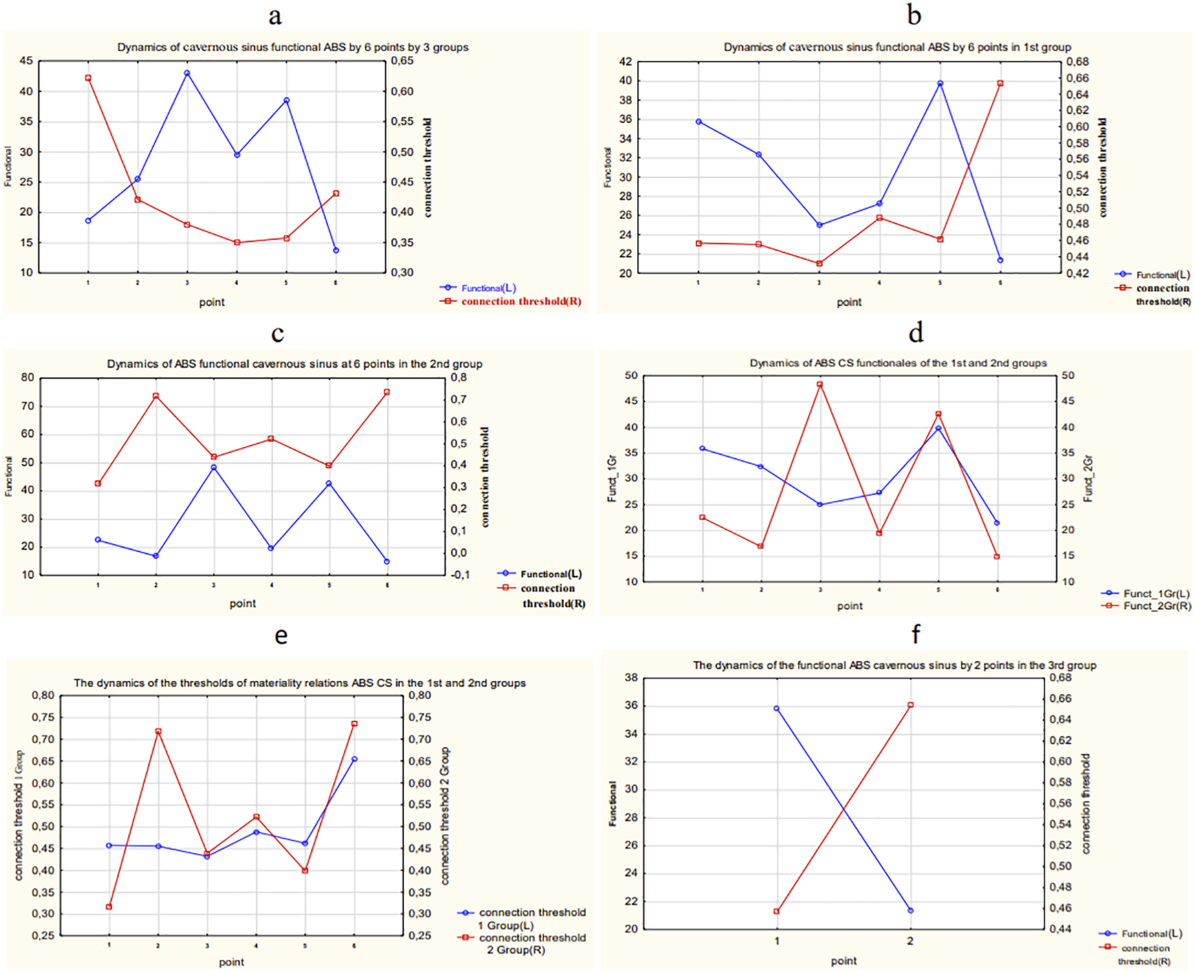
The results of calculation and comparison of the functionals in overall data and each group of patients. (A) Dynamics of functional and connection importance thresholds of the ABS parameter in a cavernous sinus in all three groups. (B) Dynamics of functional and connection importance thresholds of the ABS parameter in a cavernous sinus in the 1st group. (C) Dynamics of functional and connection importance thresholds of the ABS parameter in a cavernous sinus in the 2nd group. (D) Dynamics of functional of the ABS parameter in a cavernous sinus in the 1st and 2nd groups. (E) Dynamics of connection importance thresholds of the ABS parameter in a cavernous sinus in the 1st and 2nd groups. (F) Dynamics of functional and connection importance thresholds of the ABS parameter in a cavernous sinus in the 3rd group.

The results of calculation and comparison of the functionals in overall data and each group of patients are presented in Fig. 18.

The following conclusions about the dynamics of functionals of ABS in a cavernous sinus of patients can be made:

- The maximum shifts in the system of ABS parameters in a cavernous sinus of the 1st group of patients take place during the period between the 5th and 6th points of the research.
- The maximum shifts in the system of parameters of ABS in a cavernous sinus of the 2nd group of patients take place also during the period between the 5th and 6th points of the research, but the variation of the connection importance threshold and the function value are much higher in the 2nd group than in the 1st group. In other words, the 2nd group has more unstable dynamics of parameters of ABS.
- The 3rd group of patients has relatively small variations of the researched parameters.

## Discussion

The developed DM Core ontology that represents the knowledge of DM algorithms, characteristics, and processes is capable to answer the competency questions presented in Table 2.

**Table 2:** The evaluation of the DM Core ontology.

	Competency questions (CQ)	Answers	Correct?
CQ 1	Given data characteristics “DataWithClassImbalance”, which characteristics should the DM algorithms have so that they are “suitable for”?	ToleranceToClassImbalance	Yes
CQ 2	Given task requirements “BinaryClassification”, which output models should the processes have so that they are “available for”?	BinaryClassificationModel	Yes
CQ 3	Which characteristics does the DM algorithm “BayesianAlgorithm” have?	ToleranceToClassImbalance, HandlingOfClassificationCosts, BiasVarianceProfile	Yes
CQ 4	What outputs does the process “data mining goals identification” have?	data mining goals description, data mining success criteria description	Yes
CQ 5	Which algorithms does the process “DimensionReduction” employ?	PCA	Yes
CQ 6	How is the data characteristics “LongTSDataset” defined?	The time series dataset has length more than 700	Yes
CQ 7	What are the post processes of a process “BusinessUnderstanding”?	DataUnderstanding	Yes
CQ 8	What sub-processes does the stage “BusinessUnderstanding” have?	application objectives identification, application resources assessment, data mining goals identification	Yes
CQ 9	What parameters does SVC-Algorithm need?	CapacityParameter, KernelTypeParameter	Yes

The evaluation of the proposed ontology merging method and sub-ontology extraction method are as follows:

- In the ontology merging method, the numbers of the axioms of the initial ontologies are *N*_*a*_ and *N*_*b*_. Firstly, the axioms are extracted in time *N*_*a*_ + *N*_*b*_. Then we need to rename all the axioms in time *N*_*a*_ + *N*_*b*_. So totally, the time complexity of the ontology merging method is *O*(*N*_*a*_ + *N*_*b*_).
- In the sub-ontology extraction method, the numbers of the classes and properties of the initial ontology are *N*_*c*_ and *N*_*p*_. The extraction process makes a search of superclasses according to the properties. Thus, the time complexity of the sub-ontology extraction method is *O*(*N*_*c*_ $\cdot *N*_*p*_).

The efficiency of the proposed approach was estimated according to the defined efficiency indicators:

- Completeness—The constructed DM Core ontology provides the semantic representation for the overall stages of CRISP-DM. Users can query for suitable algorithms according to the current data characteristics and task requirements step by step.
- Performance—The proposed sub-ontology extraction method, which allows extracting the task-related ontologies from DM Core ontology, reduces the complexity of ontology querying by reducing the size of the queried ontologies. In the experiment, the average reasoner running time on the extracted ontology is 242 ms versus 648 ms on the original ontology. The average querying running time on the extracted ontology is 68 ms versus 385 on the original ontology for the same query.
- Effectiveness—With the support of the proposed approach, the workflow for the analysis of Acid-Base State of patients is constructed: we select “KNN_Imputation_ED” to fill the missing values in the raw data on the state of the patients and employ two algorithms, “average criteria functions” and “Kupershtokh–Mirkin–Trofimov algorithm,” to analyze the consistency of the organism parameters’ changes. The CF algorithm allows identifying distinctive features of ABS and characteristic changes in their state in conditions of the postoperative period for various groups of patients. The results of data processing using the constructed workflow were compared with the results of manual data processing (Lushnov et al., 2016). It was identified that the results are similar and the proposed approach can be used for solving tasks of system analyses in medical domain.

The estimation results of the proposed approach according to the criteria are summarized in Table 3.

**Table 3:** The estimation results of the proposed approach.

Estimation criteria	Metrics		Proposed approach	Implementation
Completeness	Supports CRISP-DM	Business understanding	Yes	DM ontology
		Data understanding	Yes	
		Data preparation	Yes	
		Modeling	Yes	
		Evaluation	Yes	
		Development	Yes	
Effectiveness	Whether supports the DM workflow construction for medical domain.		Yes	Ontology merging module and Query server
Performance	Reasoning time		Decreased from 648 to 242 ms.	Sub-ontology extraction module
	Query time		Decreased from 385 ms to 68 ms for the same query	

The results of the experiments prove that the proposed ontology-based approach can be used to construct effective workflows for medical data system analysis. The workflows can be constructed and used in practice by specialists in subject domains, that are not experts in DM.

## Conclusions

Aiming at the DM workflow construction in the medical domain, this article proposes an ontology based approach to support the algorithm selection for system analyses of human organism states. The main components of the proposed approach include a DM Core ontology to describe general DM knowledge; an ontology merging method to construct domain-oriented DM ontologies; a sub-ontology extraction method to reduce the complexity of ontology querying and a query server to construct the DM workflow.

With the proposed approach, we construct the workflow to analyze the consistency of the parameters’ dynamics for the ABS analysis of patients at operative measures. KNN algorithm is selected to impute the missing values. CF and functionals are selected to estimate the changes of organism parameters. According to the results, the medical conclusions are obtained.

The sphere of application of the proposed approach is not limited to medical domain. It can be also used in other domains that require usage of DM techniques for solving complex applied tasks.

## Supplemental Information

10.7717/peerj-cs.777/supp-1 Supplemental Information 1 Raw dataset.

10.7717/peerj-cs.777/supp-2 Supplemental Information 2 Edited version of the dataset with software “statistica”.

## Additional Information and Declarations

### Data Availability

The following information was supplied regarding data availability: Part of the raw data is available in the Supplemental File and the complete raw data is available at GitHub: https://github.com/mantx626/MedicalData.git.
